# Novel Quasi‐Liquid K‐Na Alloy as a Promising Dendrite‐Free Anode for Rechargeable Potassium Metal Batteries

**DOI:** 10.1002/advs.202101866

**Published:** 2021-06-25

**Authors:** Zhixin Tai, Yi Li, Yajie Liu, Lanling Zhao, Yu Ding, Ziyu Lu, Zhijian Peng, Lijian Meng, Guihua Yu, Lifeng Liu

**Affiliations:** ^1^ Clean Energy Cluster, International Iberian Nanotechnology Laboratory (INL) Avenida Mestre Jose Veiga Braga 4715‐330 Portugal; ^2^ Key Laboratory of Computer Vision and Systems (Ministry of Education), School of Computer Science and Engineering Tianjin University of Technology Tianjin 300384 China; ^3^ School of Physics Shandong University Jinan 250100 China; ^4^ Materials Science and Engineering Programme and Department of Mechanical Engineering The University of Texas at Austin Austin TX 78712 USA; ^5^ School of Science China University of Geosciences Beijing 100083 China; ^6^ Centre of Innovation in Engineering and Industrial Technology, Instituto Superior de Engenharia do Porto Instituto Politécnico do Porto Porto 4200–465 Portugal

**Keywords:** dendrite‐free electrode, K‐Na alloy, potassium metal battery, quasi‐liquid state

## Abstract

Rechargeable potassium metal batteries are promising energy storage devices with potentially high energy density and markedly low cost. However, eliminating dendrite growth and achieving a stable electrode/electrolyte interface are the key challenges to tackle. Herein, a novel “quasi‐liquid” potassium‐sodium alloy (KNA) anode comprising only 3.5 wt% sodium (KNA‐3.5) is reported, which exhibits outstanding electrochemical performance able to be reversibly cycled at 4 mA cm^–2^ for 2000 h. Moreover, it is demonstrated that adding a small amount of sodium hexafluorophosphate (NaPF_6_) into the potassium bis(fluorosulfonyl)imide electrolyte allows for the formation of the “quasi‐liquid” KNA on electrode surface. Comprehensive experimental studies reveal the formation of an unusual metastable KNa_2_ phase during plating, which is believed to facilitate simultaneous nucleation and suppress the growth of dendrites, thereby improving the electrode's cycle lifetime. The “quasi‐liquid” KNA‐3.5 anode demonstrates markedly enhanced electrochemical performance in a full cell when pairing with Prussian blue analogs or sodium rhodizonate dibasic as the cathode material, compared to the pristine potassium anode. Importantly, unlike the liquid KNA reported before, the “quasi‐liquid” KNA‐3.5 exhibits good processability and can be readily shaped into sheet electrodes, showing substantial promise as a dendrite‐free anode in rechargeable potassium metal batteries.

## Graphical abstract

A novel “quasi‐liquid” potassium‐sodium (K‐Na) alloy containing only 3.5 wt% Na is demonstrated to show outstanding electrochemical performance and possess mechanical processibility better than both bare K and liquid K‐Na alloy, holding substantial promising for use as a high‐performance anode in rechargeable potassium batteries.

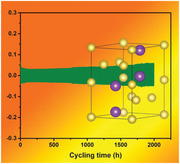

## 1 Introduction

Rechargeable alkali metal batteries (AMBs) have attracted considerable attention in the last decade given the low redox potentials of alkali metals and their high theoretical energy density that can satisfy the ever‐growing demand for electro‐mobility and renewable energy storage.^[^ ^1^, ^2^ ^]^ In particular, sodium (Na) and potassium (K) metal batteries show greater potential compared to their lithium (Li) counterparts, because Na and K are several orders of magnitude more abundant than Li and are readily accessible in oceans and rivers with a lower cost.^[^ ^3^ ^]^ The major challenges in common for all AMBs are, on one hand, to overcome the dendrite formation and growth during the metal plating, which may result in short circuit and eventually cause thermal runaway and safety problems; on the other hand, to achieve a stable electrode/electrolyte interface avoiding unfavorable side reactions and capacity decay.^[^ ^1^ ^]^ Many strategies have been proposed to address these challenges, such as microstructuring the electrode to alter the electric field distribution,^[^ ^4^ ^]^ replacing liquid electrolyte with solid or polymer electrolyte,^[^ ^5^ ^]^ engineering the solid electrolyte interphase (SEI) layer to stabilize metal re‐deposition,^[^ ^6^, ^7^ ^]^ and replacing solid electrode with liquid metal electrode.^[^ ^7^, ^8^ ^]^ In particular, liquid metals have recently drawn considerable attention for their various applications in physical chemistry, materials synthesis, and energy storage,^[^ ^9^ ^]^ and using liquid alkali metal or alloy as the battery anode is believed to be a promising solution that may intrinsically overcome the dendrite growth problem, because dendrites would be in theory unable to form at a liquid‐liquid interface.^[^ ^7^, ^10^ ^]^

For potassium metal batteries (PMBs), Goodenough and co‐workers recently proposed a K‐Na alloy (KNA) (K:Na = 66.3:33.7, w/w) anode that can remain to be liquid down to a low temperature of −12.6 °C, which showed a stable plating/stripping behavior at a low current density of 0.4 mA cm^–2^ for 2800 h.^[^ ^11^ ^]^ However, liquid KNA has very large surface tension that prevents it from wetting on ceramic solid electrolyte or a porous separator; moreover, liquid KNA cannot be directly shaped as a sheet electrode for use in batteries with a traditional sandwich architecture. To overcome this limitation, liquid KNA has been immobilized with porous carbon current collectors (e.g., carbon paper, carbon nanotube membrane) or carbon powders, in order to form a composite sheet electrode. For example, Yu's group recently demonstrated that liquid KNA could favorably wet on a graphite intercalation compound forming a homogeneous electrode, which was able to sustain stable plating and striping continuously for over 5000 h at a current density of 20 mA cm^–2^.^[^ ^12^ ^]^ Tu and co‐workers reported a non‐Newtonian state KNA by mixing liquid KNA with Super P^®^ carbon powders, which showed a low surface tension and could be painted on the surface of current collectors with good adhesion.^[^ ^13^ ^]^ Such KNA anodes were proven to be useful in stretchable energy storage devices, owing to the self‐healing properties of the liquid KNA and excellent electrochemical performance.^[^ ^13^ ^]^ Notwithstanding some progress, the flowable nature of liquid KNA makes mechanical processing of the electrodes rather difficult in practically usable AMBs (e.g., bending and winding in cylindrical/prismatic cells); moreover, the batteries might suffer from a failure or performance degradation when subjected to accidental mechanical deformation because the flowable KNA could be expelled from the porous current collector.

Herein, we report a novel “quasi‐liquid” KNA obtained by introducing only 3.5 wt% of Na into K (denoted as KNA‐3.5), which not only shows good electrochemical performance, but also possesses significantly reduced fluidity and thereby markedly improved processibility, compared to the most commonly investigated liquid KNA with a K/Na weight ratio of ≈2:1 (i.e., K:Na = 66.3:33.7, w/w), able to be easily shaped into a flexible sheet electrode. The KNA‐3.5 electrode exhibits excellent cycle stability upon repeated plating and stripping at 4 mA cm^–2^ for 2000 h without dendrite growth induced failure. Moreover, we find that by introducing a small amount of sodium hexafluorophosphate (NaPF_6_) into the potassium bis(fluorosulfonyl)imide (KFSI) electrolyte, the cyclability of both KNA‐3.5 and metallic K reference electrodes can be markedly enhanced, due likely to the formation of a “quasi‐liquid” KNA layer on the electrode surface. In situ X‐ray diffraction (XRD) investigation discloses that an unusual metastable KNa_2_ phase appears during the K and Na co‐plating process. The transient nature of KNa_2_ phase may introduce defective sites that facilitate the nucleation of K and Na and therefore their uniform deposition. When tested in a full cell using Prussian blue analogs or sodium rhodizonate dibasic as the active cathode materials, the KNA‐3.5 anode also shows much better cycle stability with respect to the bare K metal anode.

## 2 Results and Disscussion

**Figure 1:**
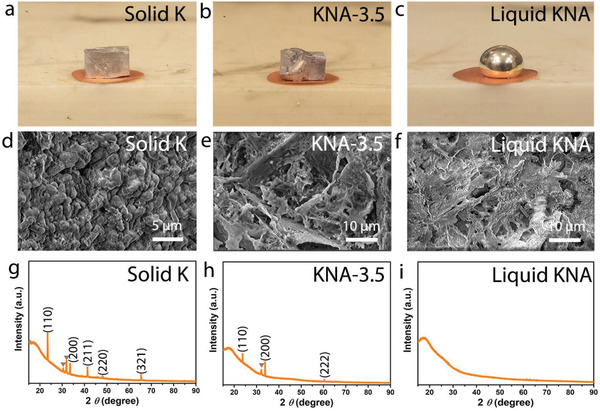
Comparison of the “quasi‐liquid” KNA‐3.5 with solid metallic K and liquid KNA‐33.7. a–c) Digital photographs showing the appearance of K and KNA on a copper foil. d–f) SEM images showing the surface morphology. g–i) XRD patterns showing the crystal structure. The yellow triangle represents the diffractions arising from K_2_CO_3_ formed upon incidental contact with air.

While previous research works on KNA were concentrated mainly on the liquid state with a typical K:Na composition of 66.3:33.7 (w/w), in this work our focus is placed on the “quasi‐liquid” state in the K‐dominant region of the K‐Na equilibrium phase diagram (Figure S1, Supporting Information), that is, the “liquid + *β*_c_ (Na in K)” phase. We prepared several KNAs with different contents of Na such as 1.8, 3.5, 5.0, and 33.7 wt% (denoted as KNA‐1.8, KNA‐3.5, KNA‐5.0, and KNA‐33.7, respectively, hereafter). Comparing to the pristine solid K metal (**Figure** **1a**), KNA‐1.8 shows little difference in terms of the appearance and ductility (Figure S2d, Supporting Information). When the Na content is increased to 3.5 wt%, the ductility is markedly improved and it becomes easier to stamp KNA‐3.5 into a desired shape (Figure 1b and Figure S2f, Supporting Information). Further increasing the Na content to 5.0 wt%, the KNA is characterized as aggregates of small particles, suggesting an increase in surface tension. When the Na content further goes up to 33.7 wt%, a liquid KNA with a contact angle of >90° is formed (Figure 1c), indicating a nonwetting nature due to the high surface tension, which is consistent with the observation in previous literature reports.^[^ ^13^ ^]^ The surface morphology of these KNAs was examined by scanning electron microscopy (SEM, Figure 1d–f). Interestingly, the KNA‐3.5 shows a surface similar to that of liquid KNA (i.e., KNA‐33.7), and the feature size of the textured surface is in the micrometer range.

The crystal structure of these KNAs and pristine K metal was investigated by XRD. The diffraction peaks (yellow triangles) at 30.4° and 31.8° were observed in the XRD patterns of all samples except the liquid KNA, which can be indexed to K_2_CO_3_ (ICDD, No. 04‐012‐7109) resulting from the incidental reaction between K and CO_2_/H_2_O in air during the XRD examination process. The pristine K metal shows diffraction peaks at 23.5°, 33.5°, 41.7°, 48.1°, and 65.5° (Figure 1g), corresponding to the (110), (200), (211), (220) and (321) crystal planes of face‐centered cubic (fcc) K (ICDD, No. 04‐004‐2201). For KNA‐3.5, the diffractions from the (211), (220), and (321) crystal planes disappear, a new diffraction peak from (222) appears, and the intensity of the principal diffraction from (110) decreases significantly, indicating that the introduction of Na into K phase changes the texture and crystallinity of metallic K. The Rietveld refinement illustrates that as a small amount of Na is introduced, the as‐obtained KNAs start to form a body‐centered cubic crystal structure with a space group of Im $\bar{3}$m (Figure S2a,c,e,g, Supporting Information). Moreover, when the Na content reaches 5.0 wt%, the diffractions from K are not visible anymore, similar to the diffraction pattern of the liquid KNA (Figure 1i), which manifests that most K has become amorphous or quasi‐liquidized (see illustration in Figure S2i, Supporting Information) compared to the pristine K (i.e., KNA‐0). The mechanical property of K metal and KNA electrodes with different Na contents was also preliminarily assessed at 50% strain. The elastic stress of the electrode reduces as the Na content in KNA increases (Figure S3, Supporting Information), suggesting that the introduction of Na into K improves the ductility of the electrode.

Given the inconvenience of liquid KNA for use to make practical PMBs, we decided to focus the investigation on the “quasi‐liquid” KNA‐3.5, which shows good processibility (Figure S3, Supporting Information) and a very low degree of fluidity. This was evidenced when placing a stainless steel weight of 23 g on a piece of KNA‐3.5 (mimicking mechanical deformation upon applying an external pressure), under which conditions the shape of the electrode was very well preserved (Figure S4, Supporting Information). In contrast, when the same weight was applied to the most commonly used liquid KNA (K:Na = 66.3:33.7, w/w), the KNA was immediately squeezed out with many flowing liquid balls on the side wall of the support. Even if the liquid KNA was immobilized in a piece of carbon cloth, when an external pressure was applied, the KNA became mobile and was detached from the carbon cloth current collector (Figure S5, Supporting Information). This suggests that liquid KNA may not be suitable for practical applications in cylindrical and prismatic cells in which the compression during cell manufacturing might cause mobilization of the liquid KNA.

The early nucleation and growth process of KNA and metallic K on Cu was investigated by SEM, using 0.8 m KPF_6_‐NaPF_6_ (K:Na molar ratio = 16.2:1) in ethylene carbonate/diethyl carbonate (EC/DEC, Sigma Aldrich, anhydrous ≥ 99.0%, 1:1 v/v) and 0.8 m KPF_6_/EC/DEC, respectively. Figure S6a–c shows the morphology of the Cu foil after K and Na electroplating at a current density of 10 μA cm^–2^ for 3, 10, and 20 h, corresponding to the nucleation capacity of 30, 100, and 200 μAh cm^–2^, respectively. The hill‐like microstructure is originated from the Cu foil current collector. We observed that K and Na were deposited on Cu with a higher density of nuclei (Figure S6a,b, Supporting Information) and formed a smooth and conformal film after 20 h (Figure S6c, Supporting Information). Moreover, when extending the plating duration to 50 h (500 μAh cm^–2^), the deposit still remained smooth and conformal, and no sign of dendrite growth was observed (Figure S7, Supporting Information). In contrast, only few K nuclei were observed at the nucleation capacity of 30 μAh cm^–2^ (Figure S6d, Supporting Information). The density of K nuclei was increased as the capacity increased up to 200 μAh cm^–2^,^[^ ^14^ ^]^ but no continuous film formed (Figure S6e,f, Supporting Information).

**Figure 2:**
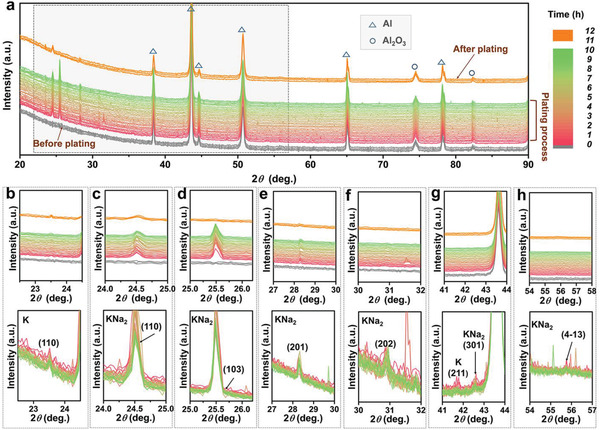
a) In situ XRD study of the KNA‐3.5 electrode during the plating process. Zoomed view in the selected 2*θ* range of b) 22.5°–24.5°, c) 24°–25°, d) 25°–26.2°, e) 27°–30°, f) 30°–32°, g) 41°–44° and h) 54°–58°. The diffraction peaks marked with triangles and circles are from the aluminum and aluminum oxide background. The patterns before and after plating were recorded without applying a plating current. Three measurements were repeated in each case.

To further scrutinize the K/Na plating process, in situ XRD examination was performed to investigate the crystal structure evolution. To do so, the KNA‐3.5 electrode was paired with an aluminum (Al) mesh (KNA‐3.5//Al), and the structure of the KNA deposited on the Al mesh was in situ monitored in a Kapton film sealed coin cell (see Experimental details, Supporting Information). 0.8 m KPF_6_‐NaPF_6_/EC/DEC was used as the electrolyte (K:Na = 16.2:1, m/m, the same as the composition in KNA‐3.5). **Figure** **2**a shows the XRD patterns acquired before, during and after the plating process. All diffraction peaks in the patterns acquired before plating can be assigned to metallic Al (ICDD, No. 01‐071‐3760) and Al_2_O_3_ (ICDD, No. 00‐004‐0787), which are marked with triangles and circles, respectively, and should result from the Al mesh electrode. As the plating process began, a number of new diffraction peaks appeared. Upon close inspection, we found that the major peaks appearing at 24.5°, 25.7°, 28.4°, 30.8°, 42.6°, and 55.8°(zoomed view in Figure 2c–h) can be indexed to the (110), (103), (201) (202), (301), and $(4\;\bar{1}\;3)$ crystal planes of the hexagonal KNa_2_ phase (ICDD, No. 00‐010‐0244), suggesting that new KNA phase formed during the plating process. In addition, weak diffractions from metallic K were also observed at 23.5° and 41.7° (Figure 2b,g), corresponding to the (110) and (211) crystal planes of fcc K (ICDD, No. 04‐004‐2201). Moreover, we also observed some irregular peaks that cannot be indexed to any polymorphs of K, Na, and KNA (e.g., the peak appearing at 31.6° in Figure 2f). These diffractions might result from the crystal planes appearing instantly during the dynamic plating process of liquid KNA. Such a phenomenon often occurs in some liquid substance,^[^ ^15^ ^]^ and has also been confirmed by modern computer simulation.^[^ ^15^ ^]^

It is worth noting that the KNa_2_ phase usually only appears at either low temperatures or high pressures (Figure S1, Supporting Information).^[^ ^16^ ^]^ This is the first time, to the best of our knowledge, that KNa_2_ phase is observed at room temperature during the electroplating of KNA in an organic electrolyte. While the formation mechanism of KNa_2_ phase under our plating conditions remains unclear and detailed investigation falls beyond the scope of the present work, we are convinced that the KNa_2_ phase may be a transient state during the dynamic reduction of K^+^ and Na^+^, because the major peak of the KNa_2_ phase (i.e., the (103) diffraction) vanished and all other diffractions from KNa_2_ became weak in intensity once the plating was stopped (see the curves on the top in Figure 2a). The metastable nature of the KNa_2_ indicates that the crystal structure of the deposit would be subjected to spontaneous re‐organization, during which a large amount of defective sites could be induced. This would facilitate K and Na nucleation, likely in an atomically layer‐by‐layer manner as reported previously.^[^ ^19^ ^]^ The dynamic metastable phase formation and re‐organization process during continuous plating may help suppress the dendrite formation and growth, enabling long‐term cycling stability.

**Figure 3:**
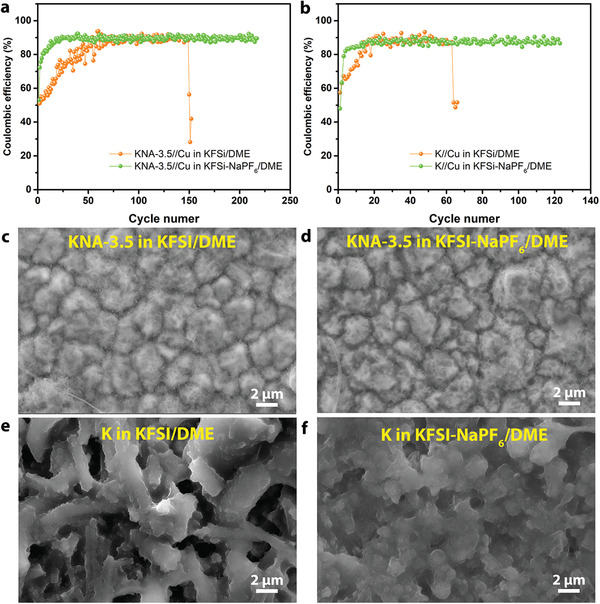
The Coulombic efficiency of a) the KNA‐3.5//Cu cell and b) the bare K//Cu cell upon repeated plating and stripping at 0.5 mA cm^–2^ in different electrolytes. After cycling, the surface morphology of the Cu counter electrode of the KNA‐3.5//Cu cell in c) KFSI/DME electrolyte and d) KFSI‐NaPF_6_/DME mixed electrolyte, and of the bare K//Cu cell in e) KFSI/DME electrolyte and f) KFSI‐NaPF_6_/DME mixed electrolyte.

The reversibility and long‐term stability of metal plating/stripping as well as the influence of electrolyte composition on the performance were further evaluated in the KNA‐3.5//Cu and bare K//Cu cells, where the two electrodes are separated by a glass fiber membrane. **Figure** **3a**,b shows the Columbic efficiency (CE, the ratio of stripping capacity to plating capacity), which is a key indicator for evalauating the reversibility,^[^ ^17^ ^]^ of the cells measured in two different electrolytes, that is, 0.4 m KFSI in dimethoxyethane (DME) and 0.4 m KFSI‐NaPF_6_ (K:Na = 16.2:1, m/m) in DME, respectively. The KNA‐3.5//Cu cell with KFSI/DME electrolyte shows an initial CE value of 51%, which then gradually increases and finally stabilizes at 89% up to 150 cycles. With the KFSI‐NaPF_6_ mixed electrolyte, the CE of the KNA‐3.5//Cu cell quickly reaches 89% and remains at this value up to ≈220 cycles. In comparison, the K//Cu cell exhibits initial and final CE values similar to those of KNA‐3.5//Cu and the metallic K embedded in the CNT scaffold reported previously,^[^ ^18^ ^]^ but having a much shorter cycle lifetime (≈60 cycles in KFSI/DME and ≈120 cycles in KFSI‐NaPF_6_/DME). Overall, we found that 1) adding a small amount of Na^+^ ions in the electrolyte can help improve the cycle lifetime and increase the CE quickly; 2) the KNA‐3.5 shows much better performance than the bare K under otherwise the same conditions. According to the above in situ XRD investigation, we believe that the presence of Na^+^ ions, whether from the electrolyte or from the stripping process of KNA‐3.5, would promote the formation of a “quasi‐liquid” KNA layer on the electrode surface, which facilitates the nucleation of K and Na, suppresses the dendrite growth and helps in the formation of a stable SEI, thereby boosting the electrochemical performance.

We further examined the surface morphology of the Cu foil of the KNA‐3.5//Cu and bare K//Cu cells after cycling. For KNA‐3.5//Cu cells tested in both KFSI/DME and KFSI‐NaPF_6_/DME, K and Na (from the stripping of KNA‐3.5 in the case of KFSI, and from both the stripping of KNA‐3.5 and NaPF_6_ in the case of mixed electrolyte) were found to deposit on the microstructured Cu conformally and no dendritic structure was observed (Figure 3c,d). In sharp contrast, a thick potassium dendrite layer was found on the surface of Cu foil in the K//Cu cell cycled in KFSI/DME (Figure 3e), and a mossy structure appeared for the cell cycled in the KFSI‐NaPF_6_/DME mixed electrolyte (Figure 3f), which can reasonably account for the comparatively shorter cycle lifetime of the K//Cu cell. These observations corroborate that the “quasi‐liquid” KNA, originating either from the electrode itself or from the electroplating, indeed contributes to the suppression of dendrite growth.

**Figure 4:**
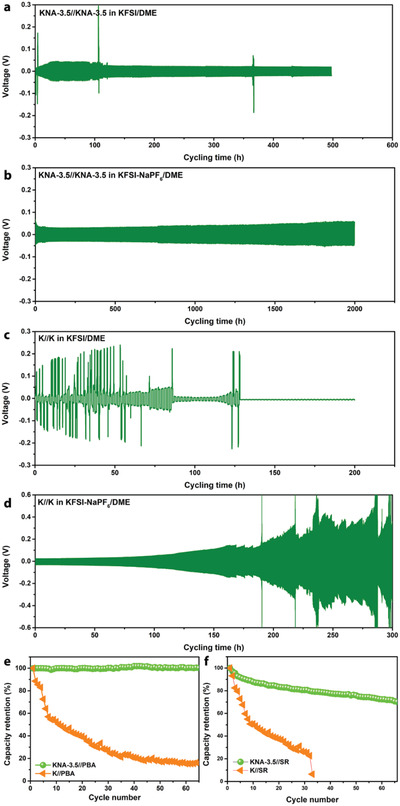
The long‐term cycling performance of KNA‐3.5 and bare metallic K electrodes measured in symmetric cells at a current density of 4 mA cm^–2^ in different electrolytes. a) KNA‐3.5//KNA‐3.5 in KFSI/DME. b) KNA‐3.5//KNA‐3.5 in KFSI‐NaPF_6_/DME. c) K//K in KFSI/DME. d) K//K in KFSI‐NaPF_6_/DME. Capacity retention of e) KNA‐3.5//PBA and f) KNA‐3.5//SR full cells cycled at 0.2 C.

Furthermore, the KNA‐3.5 and metallic K electrodes were tested and compared in symmetric cells in both KFSI/DME and KFSI‐NaPF_6_/DME electrolytes at a current density of 4 mA cm^–2^. Similar to KNA‐3.5//Cu, the KNA‐3.5//KNA‐3.5 symmetric cell also shows superior cycle reversibility and a long lifetime (**Figure** **4a**,b). In particular, it can sustain upon continuous cycling for 2000 h (4000 cycles) in the KFSI‐NaPF_6_/DME mixed electrolyte with little increase in polarization voltage. In this case, the KNA‐3.5 by itself has a “quasi‐liquid” surface, and the electrode composition would not vary markedly during cycling given that the mixed electrolyte allows for co‐deposition of K and Na. Compared to the KNA‐3.5//KNA‐3.5 cell, the K//K cell tested in KFSI/DME shows a larger polarization voltage and an unstable cycling behavior, and becomes short‐circuited rapidly (Figure 4c). Adding a small amount of NaPF_6_ helps reduce the polarization and enhance the cycle stability, but this little amount of Na^+^ ions is probably not enough to form a robust “quasi‐liquid” KNA layer that can entirely cover the electrode surface to suppress the dendrite formation and growth, such that continuous dendrite growth upon cycling would consume the electrolyte, causing significantly increased polarization (Figure 4d).

We further investigated the electrochemical performance of KNA‐3.5 in a full cell using Prussian blue analogs (PBA, 5–10 μm in size, Figure S8, Supporting Information) and sodium rhodizonate dibasic (SR, 0.3–1.5 μm in size, Figure S9, Supporting Information) as the active cathode materials given their good proven K^+^/Na^+^ storage capacity.^[^ ^19^ ^]^ The KNA‐3.5//PBA cell can deliver specific capacities of 126, 98, 65 and 32 mAh g^–1^, respectively, at the rates of 0.1, 0.2, 0.5, and 1 C (1 C = 180 mAh g^–1^ for PBA, Figure S10, Supporting Information), and the KNA‐3.5//SR cell affords specific capacities of 166, 150, 119, and 70 mAh g^–1^, respectively, at 0.1 C, 0.2 C, 0.5 C, and 1 C (1 C = 250 mA g^–1^ for SR, Figure S11, Supporting Information). Moreover, the KNA‐3.5//PBA cell exhibits 100% capacity retention after 65 charge/discharge cycles, while the bare K//PBA control shows a rapid capacity decay (Figure 4e). For the KNA‐3.5//SR cell, a slight capacity delay is observed (Figure 4f), resulting likely from the degradation of the SR cathode. Nevertheless, the cell exhibits a much better capacity retention than the bare K//SR reference cell. These results unambiguously confirm the advantage of the “quasi‐liquid” KNA anode and the KFSI‐NaPF_6_/DME mixed electrolyte.

## 3 Conclusion

In summary, we report a new quasi‐liquid KNA comprising only a small fraction of Na that has never been explored previously for use as anodes in PMBs. This novel quasi‐liquid KNA electrode not only shows extraordinary electrochemical performance, like the liquid KNA reported before, but more importantly exhibits good processibility that the flowable liquid KNA cannot offer. Our comprehensive studies reveal that the unusual metastable KNa_2_ phase appears and evanesces during plating, which facilitates the nucleation of K and Na, inhibiting the growth of dendrites and enabling the formation of a stable electrode/electrolyte interface. Moreover, adding a little amount of Na^+^ ions in K‐containing electrolyte enables the formation of a quasi‐liquid KNA layer on electrode surface and thereby markedly enhances the cycling performance. Our work opens a new avenue to the selection of bimetallic alkali metal alloys, underpinning the importance of the “quasi‐liquid” state of alloys in practical applications due to their significantly improved processibility, besides other merits dictated by the conventional liquid alloys.^[^ ^9^ ^]^ Our findings may shed new light on the development of other new high‐performance alkali metal alloy anodes.

## Conflict of Interest

The authors declare no conflict of interest.

## Supporting information

Supporting Information

## Data Availability

Research data are not shared.
