# Supplementary material for: Novel Quasi‐Liquid K‐Na Alloy as a Promising Dendrite‐Free Anode for Rechargeable Potassium Metal Batteries
Source: Adv Sci (Weinh). 2021 Jun 25;8(16):2101866. doi: 10.1002/advs.202101866 (PMC8373087; doi:10.1002/advs.202101866)
Supplement: Supplementary file 1 — Supporting Information [file ADVS-8-2101866-s001.pdf]

© 2021 Wiley-VCH GmbH

ADVANCED SCIENCE
Open Access

Supporting Information

for *Adv. Sci.*, DOI: 10.1002/advs.202101866

Novel Quasi-Liquid K-Na Alloy as A Promising Dendrite-Free Anode for Rechargeable Potassium Metal Batteries

*Zhixin Tai, Yi Li, Yajie Liu, Lanling Zhao, Yu Ding, Ziyu Lu, Zhijian Peng, Lijian Meng, Guihua Yu and Lifeng Liu,**

Supporting Information

Novel Quasi-Liquid K-Na Alloy as A Promising Dendrite-Free Anode for Rechargeable Potassium Metal Batteries

*Zhixin Tai, Yi Li, Yajie Liu, Lanling Zhao, Yu Ding, Ziyu Lu, Zhijian Peng, Lijian Meng, Guihua Yu and Lifeng Liu,**

Dr. Zhixin Tai, Dr. Yajie Liu, Ziyu Lu, Dr. Lifeng Liu

International Iberian Nanotechnology Laboratory (INL), Avenida Mestre Jose Veiga, 4715-330 Braga, Portugal

Dr. Yi Li

Key Laboratory of Computer Vision and Systems (Ministry of Education), School of Computer Science and Engineering, Tianjin University of Technology, Tianjin 300384, China

Dr. Lanling Zhao

School of Physics, Shandong University, Jinan, 250100, China

Dr. Yu Ding, Dr. Guihua Yu

Materials Science and Engineering Programme and Department of Mechanical Engineering, The University of Texas at Austin, Austin, TX 78712, USA

Ziyu Lu, Prof. Zhijian Peng

School of Science, China University of Geosciences, Beijing 100083, China

Prof. Lijian Meng

Centre of Innovation in Engineering and Industrial Technology, Instituto Superior de Engenharia do Porto, Instituto Politécnico do Porto, 4200-465 Porto, Portugal

*Corresponding author email: lifeng.liu@inl.int

Experimental details

Anode Preparation:

The K-Na alloy (KNA) was prepared by mixing metallic K and Na ingots with different weight ratios, followed by repeated press-rolling using a metal roller. The KNA was eventually shaped into a large sheet with a thickness of ~1 mm. For metallic K anodes, they were directly press-rolled out into metal sheets of ~1 mm in thickness. All the metal or alloy sheets were cut into circular disks of 10 mm in diameter for cell fabrication.

Cathode Preparation:

The Prussian blue analogues (PBAs) ($FeFe(CN)_6$ nanoparticles) were synthesized following the procedures in a previous publication (J. Mater. Chem. A 2013, 1, 10130) Sodium rhodizonate (SR) dibasic was purchased from Sigma-Aldrich (97%). The cathodes were prepared by mixing PBA powders or SR powders, super P® and polyvinylidene fluoride (PVDF) in a weight ratio of 70:20:10 in a N-methyl-2-pyrrolidone (NMP) solvent. The slurries were tape-casted on an aluminum foil and vacuum-dried for 12 h at 120 ^{o}C, and the electrodes were then cut into circular disks of 10 mm in diameter, each with a mass loading of ca. 1.20 mg cm^{-2}.

Cell Assembly:

Cells were assembled in an argon-filled glove box (GS Glovebox, Germany) with < 0.4 ppm of H_2O and < 0.1 ppm of O_2. 2032-type coin cells were used for testing in all cases. For K//Cu, KNA-3.5//Cu and KNA-3.5//Al cells, an electrolyte of 0.8 M KPF_6 or 0.8 M KPF_6-$NaPF_6$ (K/Na molar ratio=16.2:1) in ethylene carbonate/diethyl carbonate (EC/DEC, Sigma-Aldrich, anhydrous ≥ 99.0%, 1:1 v/v) were used, while for KNA-3.5//KNA-3.5 and K//K symmetric cells as well as full cells including KNA-3.5//PBA, bare K//PBA, KNA-3.5//SR and bare K//SR, the electrolyte consisted of either 0.4 M potassium bis(fluorosulfonyl)imide (KFSI) in 1,2-dimethoxyethane (DME) (Sigma-

Aldrich, anhydrous ≥ 99.5%) or 0.4 M KFSI-$NaPF_6$/DME (K/Na molar ratio = 16.2:1). The glass fiber membrane (Waterman 90 mm, 934-AHTM) was used as the separator and 120 μL electrolyte was dropped in each cell.

Materials Characterization and Electrochemical Testing:

The morphology of the electrodes was examined by scanning electron microscopy (SEM, FEI QuantaTM 650 FEG) equipped with energy-dispersive X-ray spectroscopy (EDS, Oxford INCA). X-ray diffraction (XRD) measurements including both the regular *ex-situ* and *in-situ* ones were performed on an X'Pert PRO diffractometer (PANalytical) set at 45 kV and 40 mA, using Cu K_α radiation (λ = 1.541874 Å) and a PIXcel detector. For the *in-situ* measurements, a customized holder that can accommodate an open-window coin cell was used. A piece of KNA-3.5 was utilized as the anode, and an aluminum mesh as the cathode. A Kapton film was employed as the window to allow X-rays to penetrate into the cell while protecting the metal/alloy electrode from oxidation. The crystal structure of the KNA plated on the Al mesh at 2 mA cm^{-2} was *in-situ* examined as a function of plating time. Mecchanical compression tests were conducted on cubic samples with a dimension of 7 (L) × 7 (W) × 7 (H) mm^3 (3 samples for each KNA) using a Univerisial Testing Machine (Shimadzu AGX-V Texture analyser). The specimens were compressed at a rate of 1 mm min^{-1} and the compressive depth was 3.5 mm.

The galvanostatic charge/discharge experiments were performed using a WonATech battery test station (WBCS3000S1) for the K//Cu, KNA-3.5//Cu and all cells, and a Neware battery tester (BTS4000-5V10mA) for K//K and KNA-3.5//KNA-3.5 symmetric cells. Cyclic voltammetry (CV) and chronopotentiometry were conducted on a Biologic VMP3 electrochemical workstation.

Supporting Figures

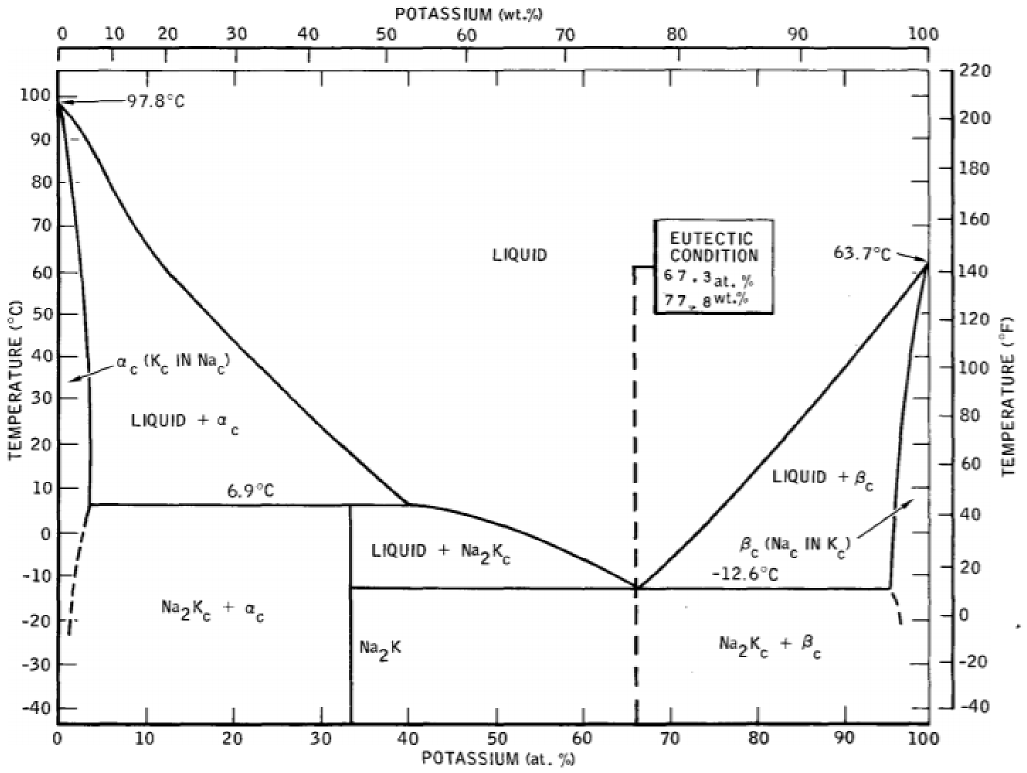

Figure S1. Sodium-potassium equilibrium phase diagram (Foust, O J. Sodium-NaK Engineering Handbook. VOLUME I. Sodium Chemistry and Physical Properties. United States: N. p., 1972. Data Source: https://www.osti.gov/biblio/4631555-sodium-nak-engineering-handbook-volume-sodium-chemistry-physical-properties).

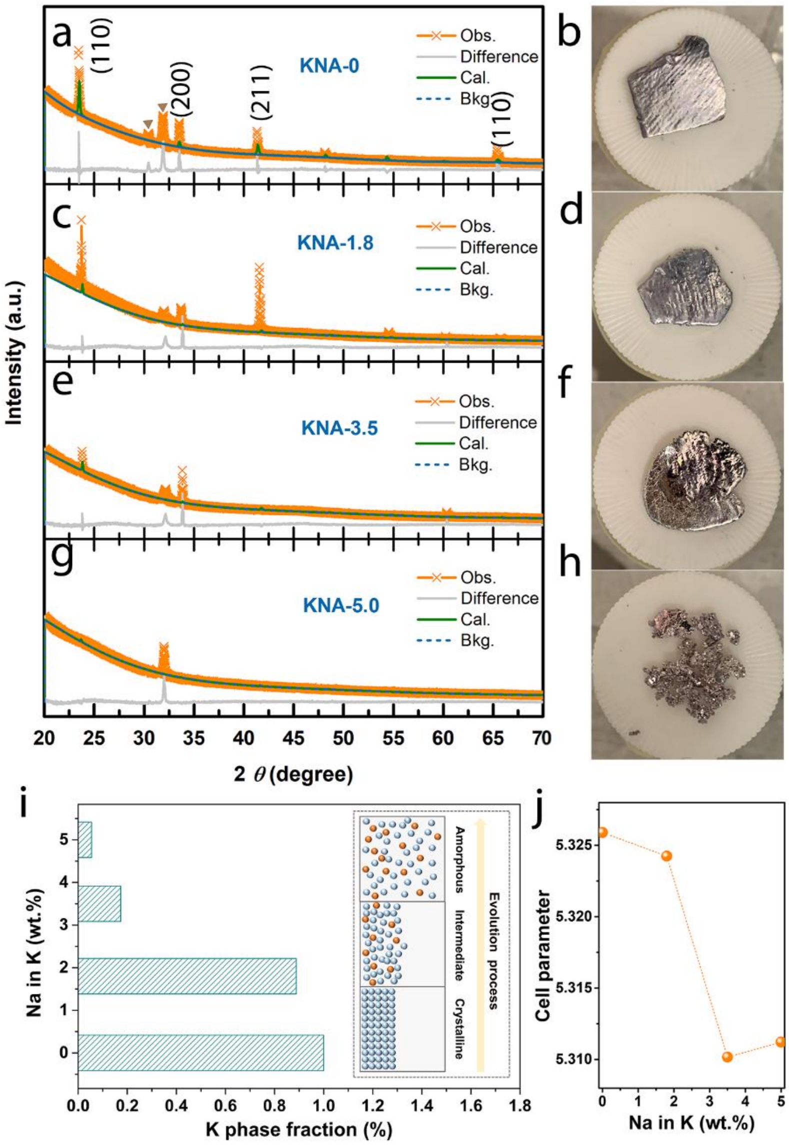

Figure S2. The phase evolution of potassium when alloying with different amounts of sodium. XRD Rietveld refinement of the KNA with (a) 0 wt.%, (c) 1.8 wt.%, (e) 3.5 wt.% and (g) 5.0 wt.% of Na. Obs. – experimental data; Cal. – calculated data; Difference – the difference between the experimentally measured and simulated patterns; Bkg. – background. The yellow triangle represents the diffraction from K_2CO_3. (b, d, e, h) the corresponding digital photographs showing the appearance of K and the resulting KNA. (i) The evolution of K phase fraction as a function of Na content (metallic K is set to 100%) and schematic illustration of the evolution process of the crystalline K upon introducing Na, i.e., from crystalline to amorphous. (j) The evolution of lattice parameters of the KNA with different Na contents.

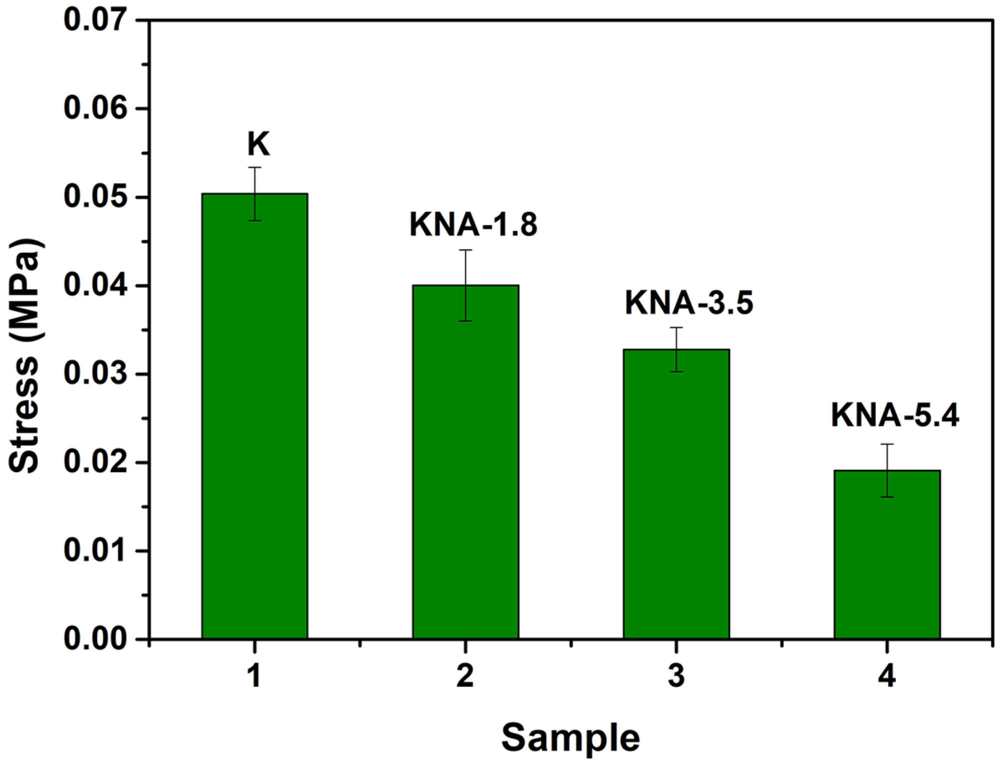

Figure S3. Compressive stress at the strain of 50% for the K metal and its alloy.

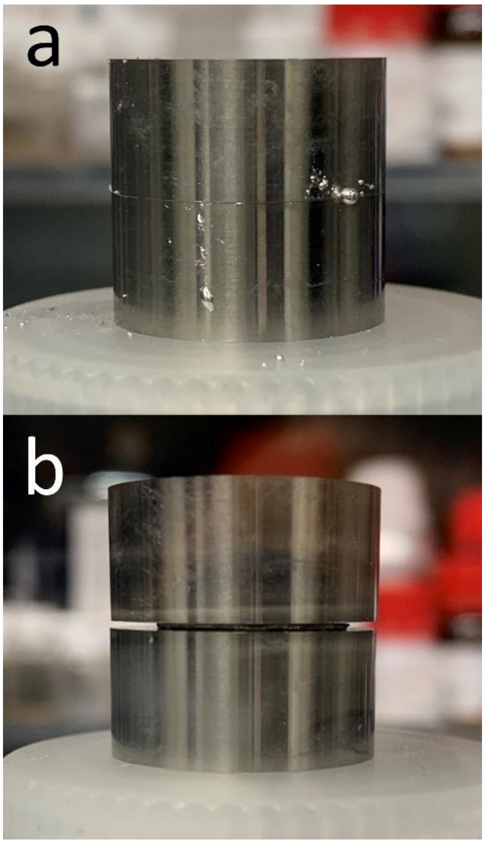

Figure S4. The comparison of the fluidity of (a) liquid KNA with a K/Na weight ratio of 66.7:33.3 (i.e. KNA-33.7) and (b) quasi-liquid KNA with a K/Na weight ratio of 100 : 3.5 (i.e. KNA-3.5) under the same loading weight of 23 g. The liquid KNA spread immediately upon loading the weight and extra liquid alloy was squeezed out, while the quasi-liquid KNA remained in its original shape.

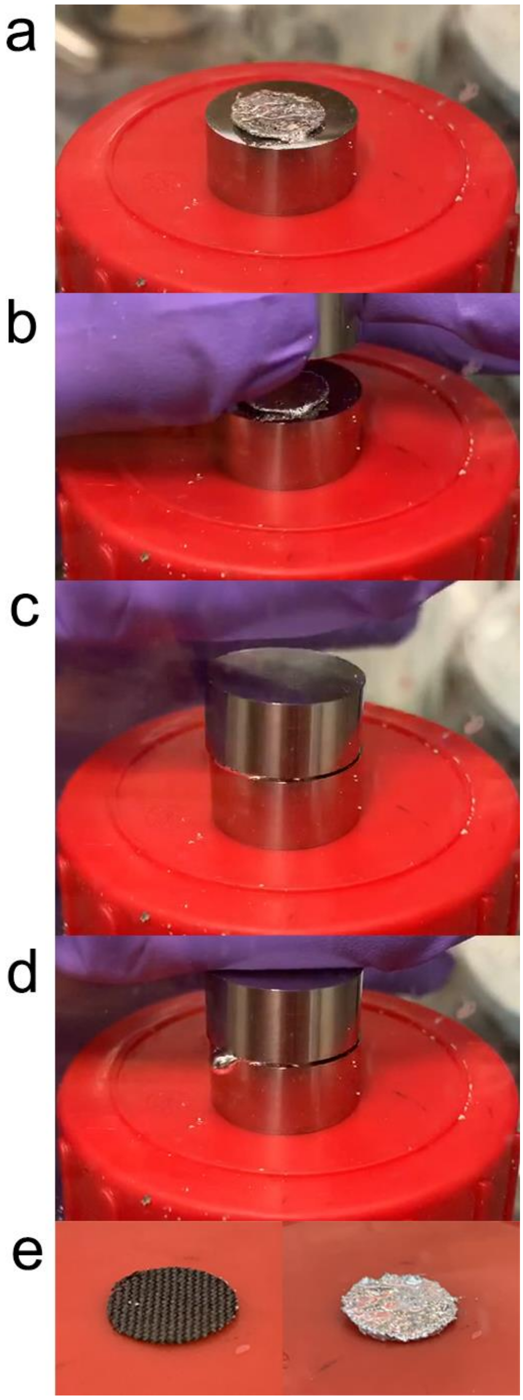

Figure S5. (a-d) Digital photographs showing the successive process of how the liquid KNA (i.e. KNA-33.7) loaded in a carbon cloth current collector was squeezed out after applying an external load (mimicking the mechanical deformation of the electrode). Digital photographs showing the appearance of (e) left: the raw carbon cloth, and right: a piece of carbon cloth absorbed with liquid KNA.

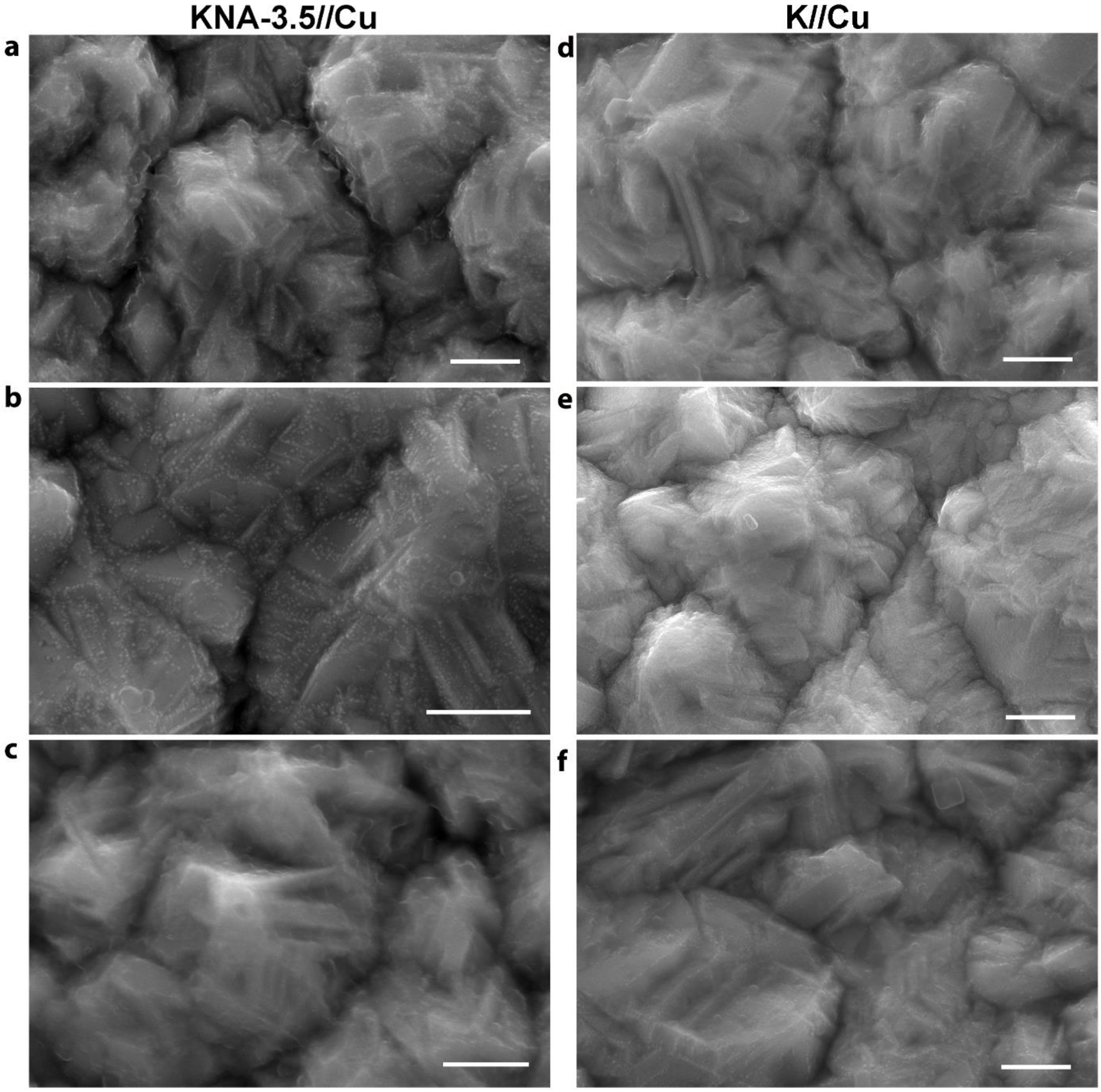

Figure S6. SEM images showing the initial nucleation and growth process of (a-c) KNA in KPF_6-$NaPF_6$/EC/DEC and (d-f) K in KPF_6/EC/DEC on a bare Cu foil upon the application of a plating current density of 10 μA cm^{-2}. Scale bars: 1 μm.

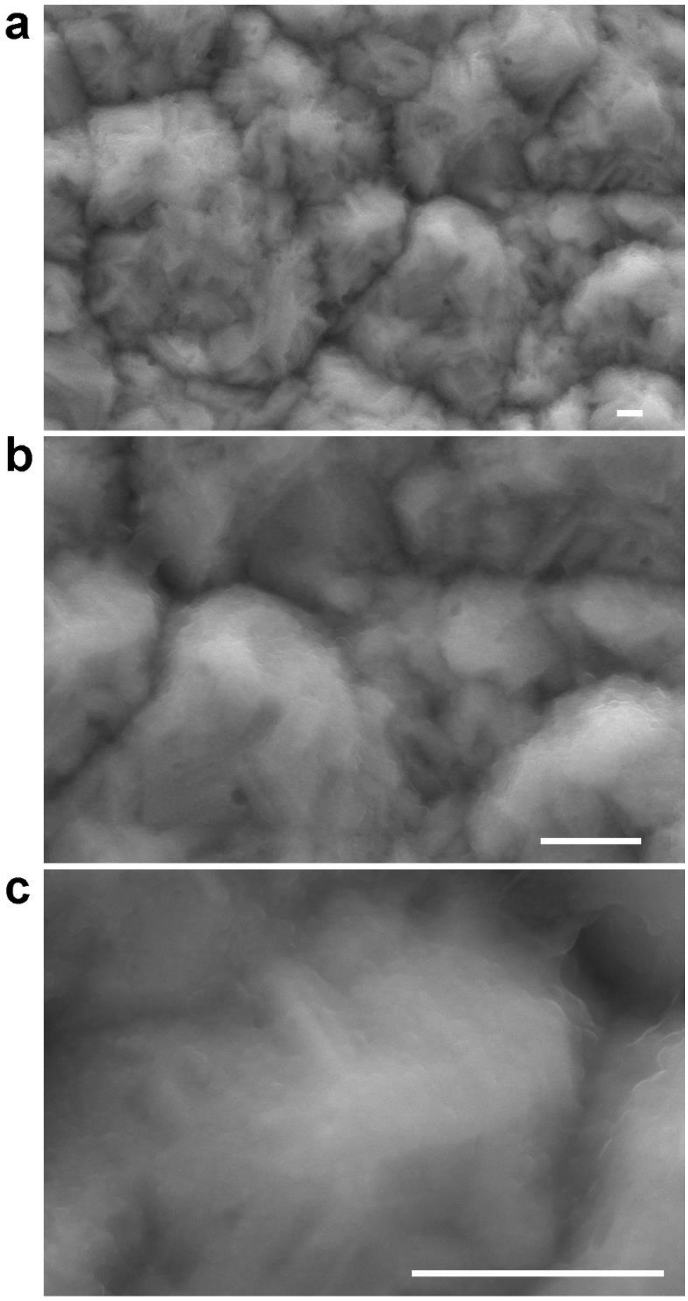

Figure S7. The morphology of the Cu foil after K/Na plating in the KNA-3.5//Cu cell at a current density of 10 µA cm^{-2} with the nucleation capacity of 500 µAh cm^{-2} (50 h), taken at different magnifications (a-c). The electrolyte is KPF_6-$NaPF_6$/EC/DEC.

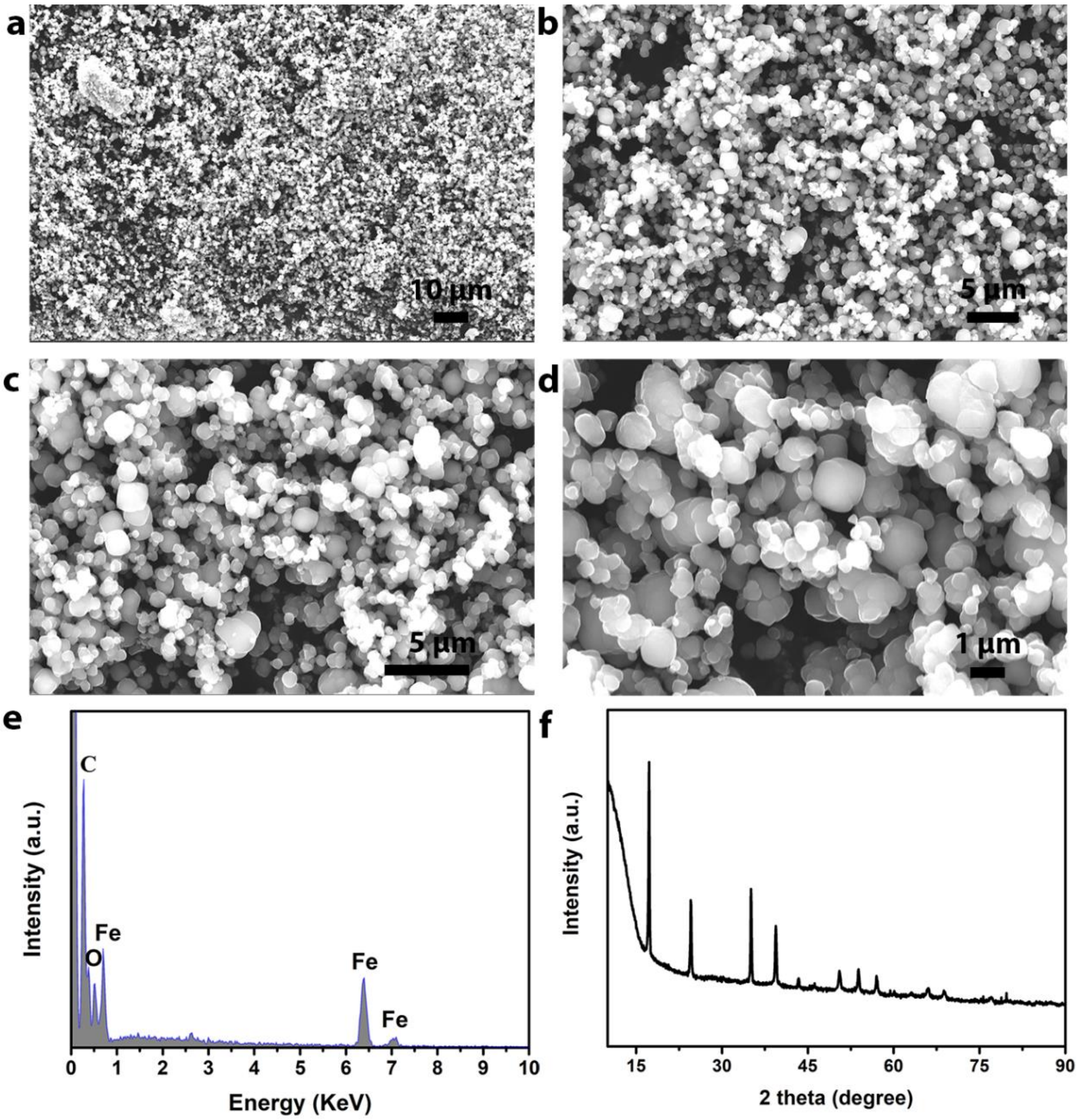

Figure S8. Morphology, composition and crystal structure characterization of Prussian blue analogue (PBA) electrode materials. (a-d) SEM images showing the morphology. (e) EDS spectrum. (f) XRD pattern.

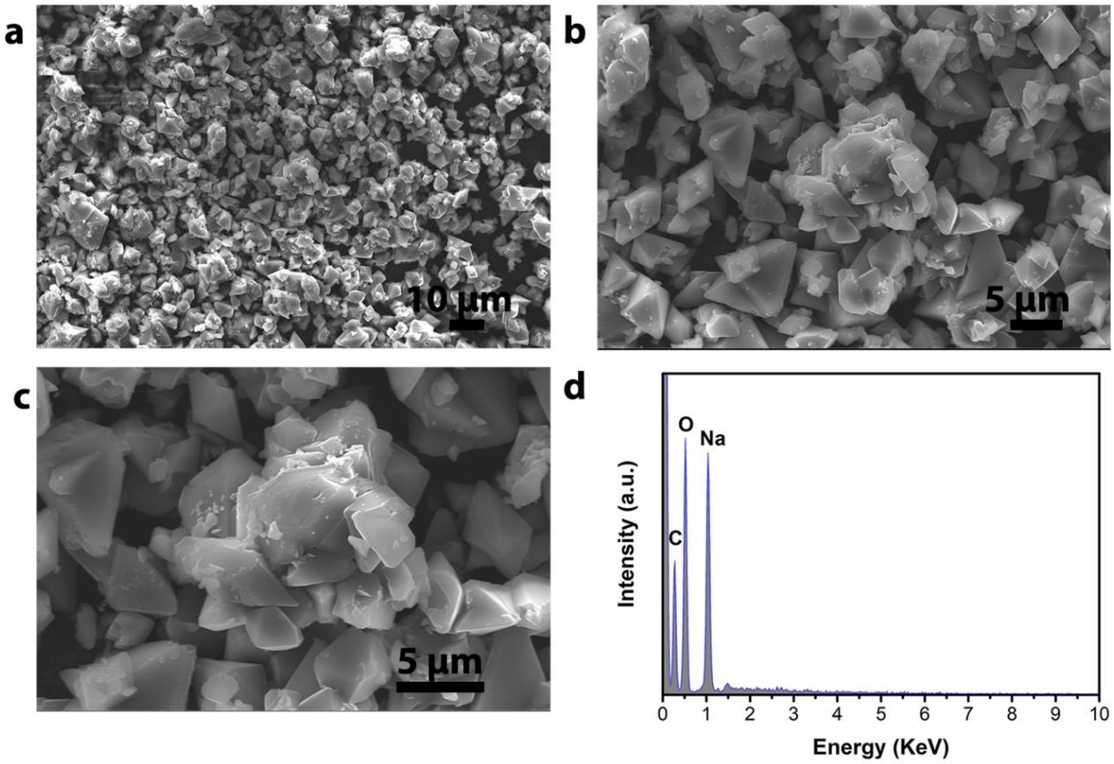

Figure S9. Morphology and composition of sodium rhodizonate dibasic (SR) cathode materials. (a-c) SEM images taken at different magnifications. (d) EDS spectrum.

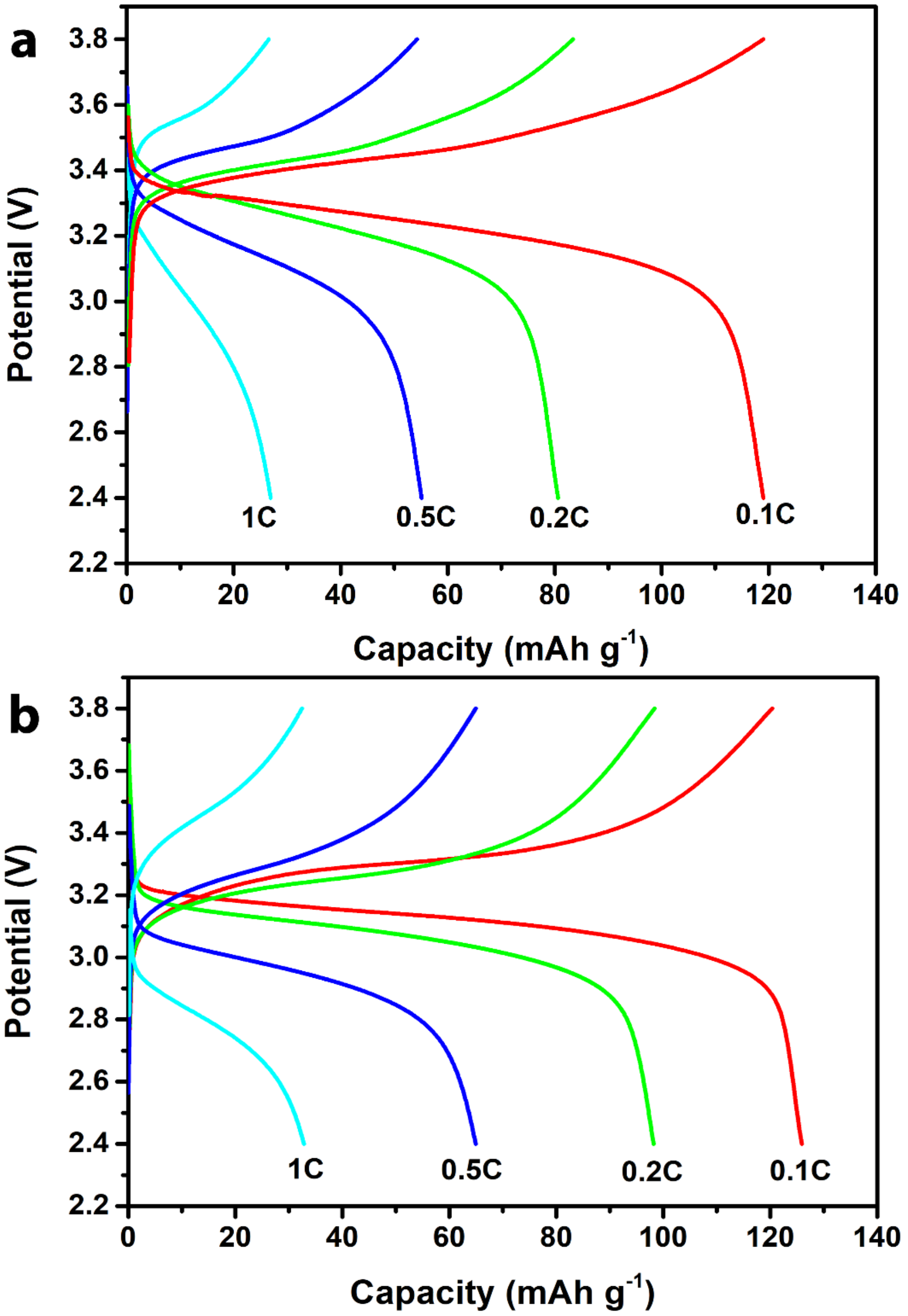

Figure S10. The charge/discharge profiles at the different rates for (a) K//PBA and (b) KNA-3.5//PBA full cells.

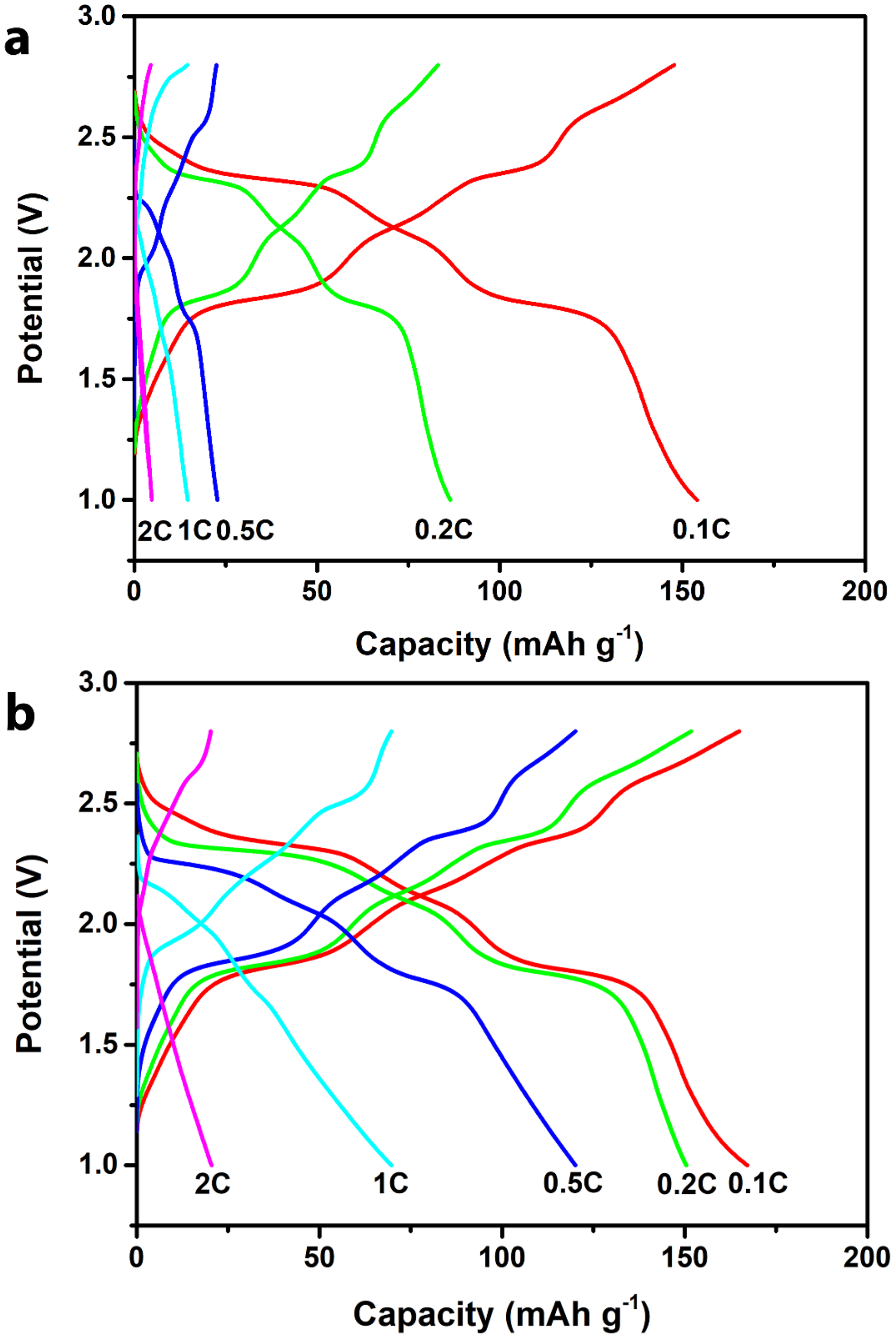

Figure S11. The charge/discharge profiles at the different rates for (a) K//SR and (b) KNA-3.5//SR full cells.
